# Transcriptional sequencing analysis reveals the potential use of deer antler for “tonifying the kidney and strengthening bone”

**DOI:** 10.1186/s13018-022-03308-w

**Published:** 2022-09-14

**Authors:** Zhenwei Zhou, Tiancheng Wang, Yuyan Jia, Tingting Wang, Enjing Luo, Jinghong Zhong, Jingcheng Zhang, Jianyu Wang, Yuchi Wei, Daqing Zhao, Baojin Yao

**Affiliations:** 1 Jilin Ginseng Academy, Changchun University of Chinese Medicine, Changchun, 130117 Jilin China; 2 College of Traditional Chinese Medicine, Changchun University of Chinese Medicine, Changchun, 130117 Jilin China

**Keywords:** Deer antlers, Pharmaceutical mechanism, Chronic kidney disease, Osteoporosis, Kidney governing bone

## Abstract

**Background:**

It is recorded in the Chinese Pharmacopoeia that deer antlers can be used to tonify the kidney and strengthen bone. Although numerous studies have demonstrated that deer antler has protective effects on the kidney and bone, its molecular mechanisms remain to be elucidated. The aim of this study was to explore the molecular mechanism underlying its effects on the bone and kidney.

**Methods:**

Water extract of pilose antler was prepared and then filtered through a 0.45 μm Hollow Fiber Cartridge (GE Healthcare, USA). The filtrate was freeze-dried by a Heto PowerDry LL3000 Freeze Dryer (Thermo, USA) and stored at − 80 °C. Rats were treated with deer antler extract (DAE) prepared in advance, and gene regulatory network in the kidney and bone was detected by RNA-Seq technique. Micro-CT was used to detect bone trabecular formation, bone mineral density (BMD) and bone volume fraction (BV/TV).

**Results:**

The results demonstrate that DAE could jointly heighten renal function by maintaining renal homeostasis, combating renal fibrosis, and reducing renal inflammation by regulating ion transport. Furthermore, DAE can strengthen the bone system by stimulating osteoblast differentiation and regulating bone regeneration and the bone marrow microenvironment. Micro-CT results confirmed that DAE can promote bone trabecular formation and increase BMD and BV/TV. We also identified many genes that can regulate both the kidney and bone simultaneously, which explained the theory of “kidney governing bone” at the molecular level and provided possible strategies for further application of this theory to treat diseases.

**Conclusions:**

DAE enhances renal function, maintains renal homeostasis, positively regulates skeletal system development, and increases bone mineral density. The underlying mechanism involves improving the expression levels of functional genes involved in renal function and regulation and repair, as well as genes that positively regulate skeletal system development.

**Supplementary Information:**

The online version contains supplementary material available at 10.1186/s13018-022-03308-w.

## Introduction

Deer antlers are extraordinary organs; stem cells in antlers can maintain their complete regeneration, which is incomparable to any other mammal. The pharmacological activities are mainly attributed to the major bioactive compounds, amino acids, polypeptides, and proteins [1]. Deer antlers have long been used medicinally to treat kidney and bone-related conditions. Modern pharmacological studies have shown that deer antlers can protect against acute kidney injury and osteoporosis [2, 3]. According to the theory of traditional Chinese medicine, there is a strong connection between the kidney and the bone. The kidney stores essence, governs bones, and generates marrow. Sufficient kidney essence ensures powerful bones, whereas a deficiency of kidney essence results in brittleness of the bones. Bone mass is determined by kidney defect or hyperplasia, and this correlation has been demonstrated [4–7].

Chronic kidney disease (CKD) is a progressive disease with no cure and high morbidity and mortality, which occurs most commonly in the general adult population, especially in people with diabetes and hypertension. CKD is the 16th leading cause of years of life lost worldwide, and changes associated with nephropathy include glomerular hypertrophy, glomerulosclerosis, tubulointerstitial inflammation, and fibrosis [8, 9]. Additional pharmacol ogical interventions and the development of innovative strategies are necessary to ensure optimal kidney-preserving care and to achieve greater longevity and better health-related quality of life for these patients.

Osteoporosis is a metabolic bone disease characterized by low bone density and deterioration of bone architecture, leading to an increased risk of fractures. Osteoporosis can be caused by many factors, including aging, postmenopausal status, vitamin D deficiency, and low calcium intake [10]. This asymptomatic condition often goes undiagnosed until it manifests as a fracture. Anti-resorptive drugs, such as bisphosphonates and the RANKL inhibitor denosumab, are currently the most widely used osteoporosis medications. Despite remarkable advances, concerns remain about the rare side-effects of anti-resorptive drugs, particularly bisphosphonates [11, 12].

In this research, the gene expression patterns in the kidney and bone of Sprague–Dawley (SD) rats after DAE treatment were revealed by RNA-Seq. We demonstrated that DAE increased kidney function and bone density, likely by maintaining renal homeostasis, combating renal fibrosis, and reducing renal inflammation by regulating ion transport. In the skeletal system, DAE strengthens the bone system in diverse ways, such as by stimulating osteoblast differentiation, and regulating bone regeneration and the bone marrow microenvironment. Simultaneously, we identified multiple genes that regulate both the kidney and bone. These results suggest that DAE can be used to treat kidney and skeletal system diseases and accord with the theory of “kidney governing bone” and provide a genetic explanation for the “kidney governing bone” theory, which has been widely used in clinical practice for thousands of years.

## Materials and methods

### DAE preparation

The antlers of three 4-year-old Chinese Sika deer in the rapid growth period (60 days) reared in the Shuangyang deer farm of Changchun, China were collected. The antlers were rinsed to cleanliness with pre-cooled MilliQ water and subsequently homogenized with a high-speed tissue homogenizer (Voshin, China). The homogenate was centrifuged at 12,000×*g* at 4 °C for 30 min. The supernatant was filtered through a 0.45 μm Hollow Fiber Cartridge (GE Healthcare, Chicago, IL). The filtrate was freeze dried by a Heto PowerDry LL3000 Freeze Dryer (Thermo, Waltham, MA) and stored at − 80 °C.

### Laboratory animal treatment

Male Sprague–Dawley (SD) rats, weighing 200–250 g (*n* = 20), were purchased from the Changchun Yisi Laboratory Animal Technology Co. Ltd. (Changchun, China). The animals were supported in a room with a standardized condition (22 ± 2 °C, 50% ± 10% humidity, 12 h light/dark cycle). Rats were acclimatized for 7 days with free access to water and standard rat nutrients before the experiments. SD rats were then randomly divided into two groups (10 rats per group). DAE (0.2 g/kg/d) was orally administered to rats in the experimental group daily for 3 consecutive weeks, while those in the control group received drinking water. The dose of DAE selected for use in the animal experiment was calculated based on normalization to interspecies differences in body surface area. All animal experimental procedures were performed in accordance with corresponding standards and guidelines, and approved by the Institutional Animal Care and Use Committee of Changchun University of Chinese Medicine (No. ccucm-2017-0015).

### Extraction of tissue RNA and illumina sequencing

The rat tissues were collected after 21 days of intragastric administration. After the rats were euthanized, the femurs and kidneys of each group were removed and submerged in pre-cooled phosphate buffered saline (PBS) to remove the remaining tissues, and the clean femurs and kidneys were ground in liquid nitrogen. Total RNA was extracted with TRIzol reagent (Invitrogen, USA) according to the manufacturer’s protocol. The RNA integrity number (RIN) was calculated using an Agilent 2100 Bioanalyzer (Agilent Technologies, USA) to determine the RNA quality. Paired-ended mRNA libraries were prepared using the Tru-Seq Stranded mRNA kit (Illumina, USA) according to the manufacturer’s protocol. Transcriptome sequencing by RNA-Seq was conducted on an Illumina HiSeq 2500 platform (Illumina, USA).

### RNA-Seq data analysis

RNA-Seq was filtered to remove low-quality reads and adaptor sequences to obtain clean readings. The clear reads of each sample were plotted on the reference genome of the rat (Rattus norvegicus) using HISAT [13]. FPKM algorithm calculation was performed to detect gene expression levels [14]. BLAST was used to perform annotations by searching for each sequence against the following databases: Nr, Nt, Swissprot, Gene Ontology (GO), and Kyoto Encyclopedia of Genes and Genomes (KEGG). Genes with a log2 fold change of ≥ 1 or ≤ 1 and a *p*-value ≤ 0.001, as guided by the DEGseq R package, were considered to be differentially expressed [15].

### Function and pathway enrichment analysis of differentially expressed genes

GO and KEGG enrichment analysis was conducted with the R function phyper. The hypergeometric test and Bonferroni correction were applied in the enrichment analysis. After multiple testing corrections, the GO terms or pathways with a corrected *p*-value (*Q* value) of < 0.05 were considered significantly enriched in the differentially expressed genes [16].

### Quantitative real-time PCR validation of gene expression levels

qRT-PCR was used to validate the expression levels of the differentially expressed genes identified by RNA-Seq analysis. Briefly, total RNA was extracted with TRIzol reagent (Invitrogen, USA) according to the manufacturer’s protocol. cDNA was synthesized using the iScript cDNA Synthesis Kit (Bio-Rad, USA) and amplified using SsoAdvanced Universal SYBR® Green Supermix (Bio- Rad, USA) on a CFX Connect Real-Time PCR Detection System (Bio-Rad, USA) under standard amplification conditions. The gene expression levels were normalized to the *RPL4* and calculated using the 2^−ΔΔCT^ method [17].

### Micro-CT measurement of bone parameters

Samples were scanned using a Skyscan 1174 benchtop micro-CT (50 kV, 800 μA). The scan resolution was 14.5 μm, and the field of view was 1304 × 1024. A total of 125 consecutive slices of the femoral epiphyseal plate, including a 1.8 mm thick medullary cavity, were used to image the three-dimensional reconstructed region of interest. The images were reconstructed by N-Recon software; Various parameters including BMD and BV/TV were measured by CT-AN software.

## Results

### RNA-Seq, transcriptome assembly, and functional annotation

The transcriptomes of kidney and bone from rats with or without the treatment with DAE were separately sequenced using a paired-end Illumina sequencing method. All read sequences were deposited in the NCBI Sequence Read Archive (SRA) database under accession numbers PRJNA632645 and PRJNA631858. After removing low-quality reads and adapter sequences, 56,703,256 and 52,841,234 clean reads were obtained from the kidney of untreated rats (blank), whereas 47,030,076 and 44,033,792 were obtained from the bone of DAE-treated rats, as shown in Table 1. The quality assessment showed that the Q30 percentages were > 91%, and the GC content percentages were approximately 50%. For the blank and DAE-treated kidney samples, 52,937,746 and 49,154,050 reads were mapped to the rat genome, respectively. In total, 13,051 of 15,767 (blank) and 12,929 of 15,638 (DAE) transcripts were annotated against the non-redundant (NR) NCBI protein database and the Swiss-Prot database, respectively. For the blank and DAE-treated bone samples, 43,821,554 and 41,234,712 reads were mapped to the rat genome, respectively. In total, 12,806 of 15,648 (blank) and 12,666 of 15,427 (DAE) transcripts were annotated against the non-redundant (NR) NCBI protein database and the Swiss-Prot database, respectively.

**Table 1:** Statistics for the sequencing and assembly results

Statistics	Kidney		Bone	
	Blank	DAE	Blank	DAE
Clean reads	56,703,256	52,841,234	47,030,076	44,033,792
Q30 percentage	91.71	91.49	92.76	92.92
GC percentage	51.12	49.97	51.82	50.87
Total mapped reads	52,937,746	49,154,050	43,821,554	41,234,712
Total transcripts	15,767	15,638	15,648	15,427
Known transcripts	13,051	12,929	12,806	12,666

### Comparative analysis of differentially expressed genes

For the blank and DAE-treated kidney samples, the differential expression analysis identified 1665 genes that were significantly differentially expressed between the DAE-treated and blank groups (log2 fold change ≥ 1 or ≤ − 1 and *p* ≤ 0.001), including 680 upregulated genes and 985 downregulated genes (DAE vs. blank). For the blank and DAE-treated bone samples, the differential expression analysis identified 607 genes that were significantly differentially expressed between the DAE-treated and blank groups (log2 fold change ≥ 1 or ≤ − 1 and *p* ≤ 0.001), including 131 upregulated genes and 476 downregulated genes (DAE vs. blank), as shown in Table 2.

**Table 2:** Statistical analysis of differentially expressed genes in kidney and bone (DAE vs. Blank)

Statistics	Number
*Kindey*	
Differentially expressed mRNAs	1665
Upregulated mRNAs	680
Downregulated mRNAs	985
*Bone*	
Differentially expressed mRNAs	607
Upregulated mRNAs	131
Downregulated mRNAs	476

### GO and KEGG enrichment analysis of differentially expressed genes in the kidney and bone under DAE treatment

**Fig. 1:**
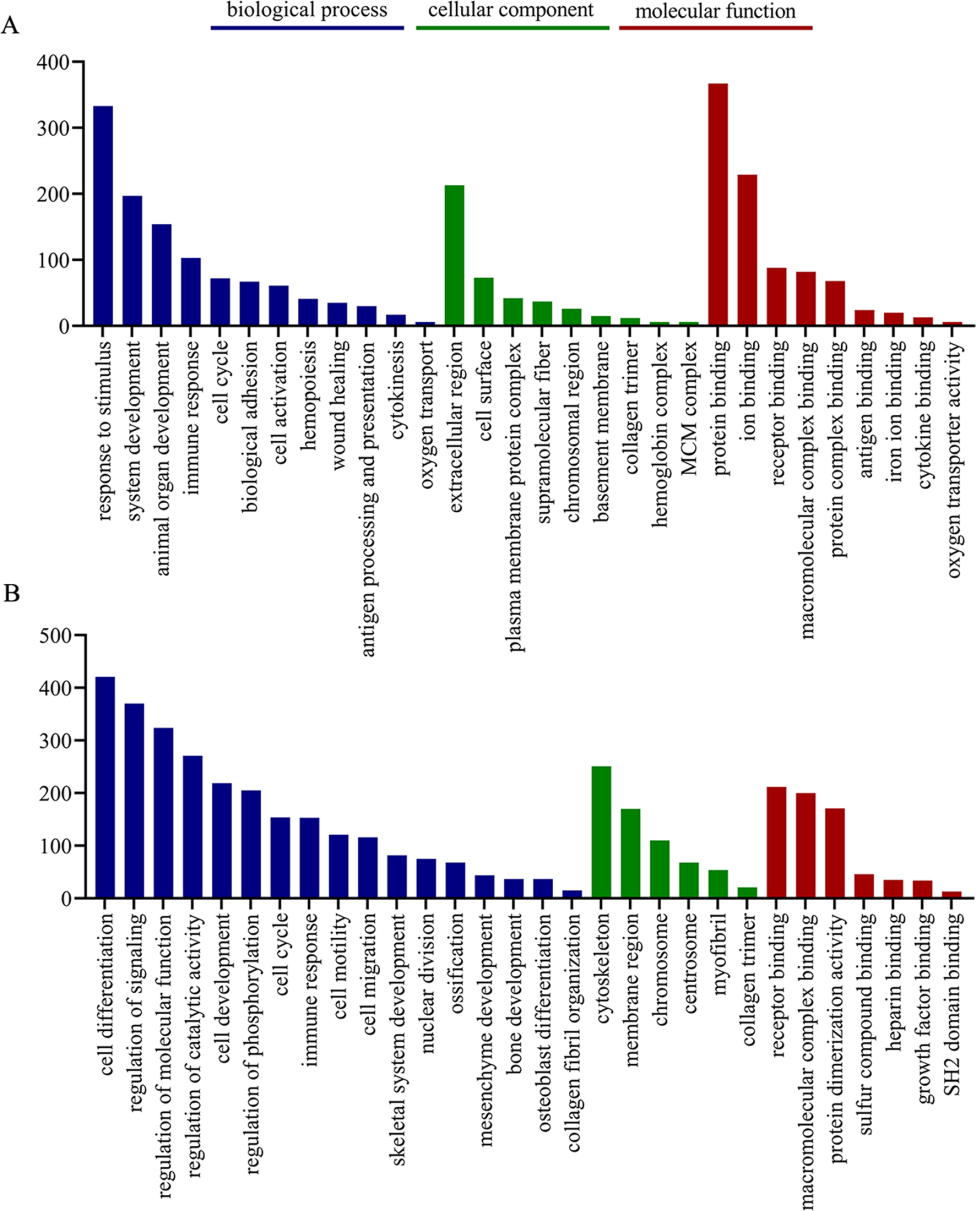
GO enrichment analysis of differentially expressed genes in bone and kidney under DAE treatment. **A** Bone. **B** Kidney. The x-axis indicates the number of genes in a category. The y-axis indicates the significantly (*p* < 0.05) enriched GO terms in the categories of biological process, molecular function, and cellular component

GO enrichment analyses were performed to gain insight into the differentially expressed genes involved in kidney and bone functions under DAE treatment, as shown in Fig. 1. For the kidneys, the significantly enriched GO terms related to biological processes were mainly involved in the categories of response to stimulus or system development, animal organ development, and immune response; the significantly enriched GO terms related to cellular component were mainly involved in the categories of extracellular region, cell surface, and plasma membrane protein complex; and the significantly enriched GO terms related to molecular function were mainly involved in the categories of binding and oxygen transporter activity. For bone, the significantly enriched GO terms related to biological processes were mainly involved in the categories of cell differentiation, regulation of signaling, and regulation of molecular function; the significantly enriched GO terms related to cellular component were mainly involved in the categories of cytoskeleton and membrane region and chromosome; and the significantly enriched GO terms related to molecular function were mainly involved in the categories of binding and protein dimerization activity.

**Fig. 2:**
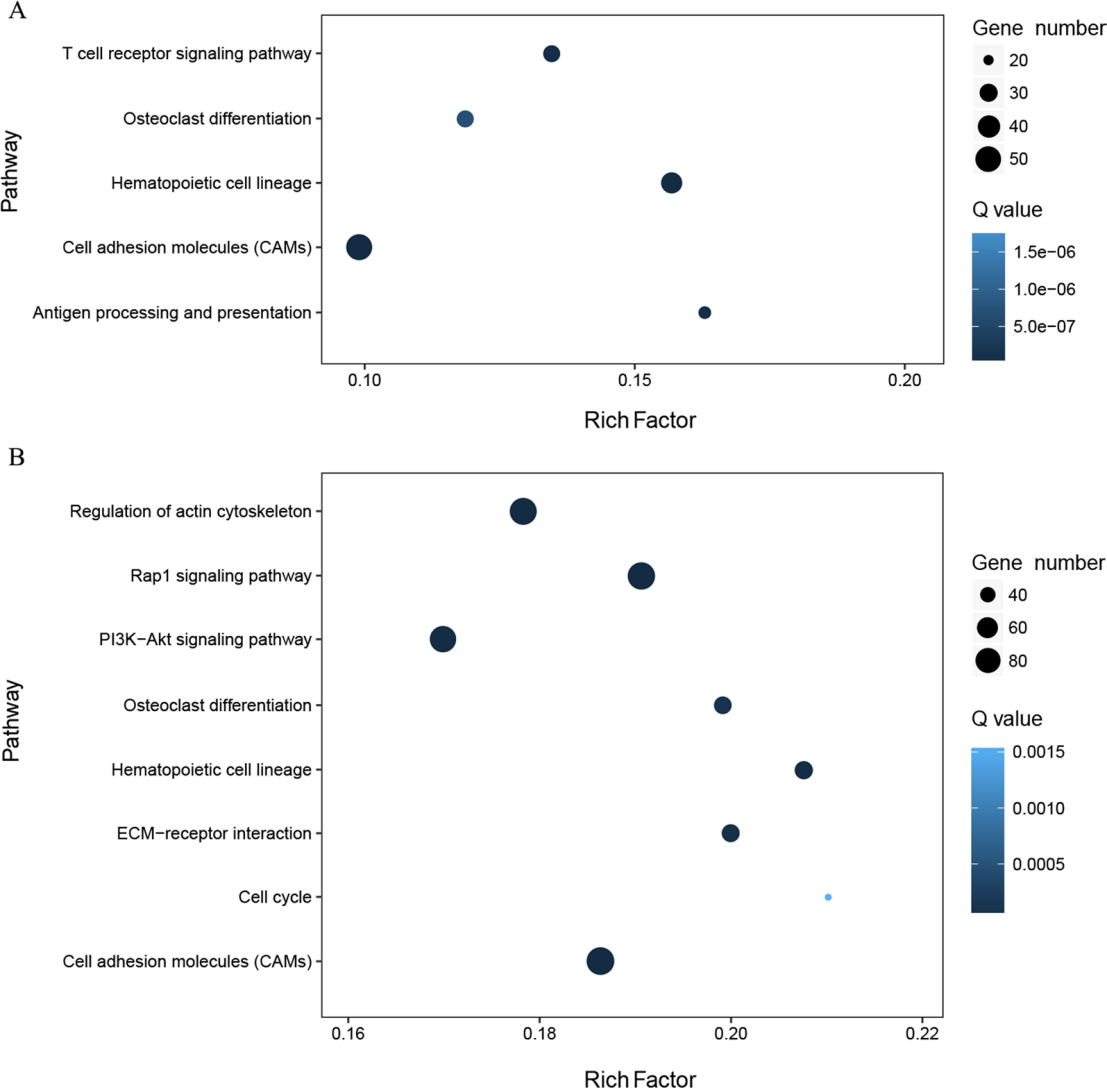
KEGG enrichment analysis of differentially expressed genes in bone and kidney under DAE treatment. **A** Bone. **B** Kidney. The x-axis represents rich factor, which is the ratio of the number of target genes divided by the number of all the genes in each pathway, and the y-axis represents the enriched pathway. The size and color of the dots represent the gene number and the range of *Q* values, respectively

KEGG pathway enrichment analyses were performed to further explore the possible physiological processes and pathways of these differentially expressed genes involved in kidney functions and bone under DAE treatment, as shown in Fig. 2. For the kidney, the significant enriched pathways were mainly involved in the categories of cell adhesion molecules, hematopoietic cell lineage, the T cell receptor signaling pathway, osteoclast differentiation, and antigen processing and presentation. For the bone, the significantly enriched pathways were mainly involved in the categories of cell adhesion molecules, the regulation of the actin cytoskeleton, the Rap1 signaling pathway, the PI3K-Akt signaling pathway, osteoclast differentiation, hematopoietic cell lineage, ECM-receptor interaction, and the cell cycle.

### DAE increases the expression level of genes involved in renal function and regulates renal homeostasis

CKD is influenced by many factors that seriously affect the quality of life of patients. To explore the molecular mechanism of the protective effect of DAE on the kidney, we analyzed multiple gene expression levels. As shown in Table 3, the expression levels of *Slco1a6, Resp18, Tmigd1, Rbm3, Azgp1, Lpl, Casr, Slco4c1,* and *Bmpr1b* were significantly upregulated, which are involved in the positive regulation of renal function and homeostasis.

**Table 3:** The expression levels of genes related to kidney function (DAE vs. Blank)

Gene name	Blank (FPKM)	DAE (FPKM)	log2 fold change (DAE/Blank)	*p* value
Solute carrier organic anion transporter family member 1A6 (Slco1a6)	88.44	188.27	1.09	6.00E−241
Regulated endocrine-specific protein 18 (Resp18)	56.53	114.99	1.02	2.39E−33
Transmembrane and immunoglobulin domain-containing protein 1 (Tmigd1)	56.81	114.03	1.00	7.68E−66
RNA-binding protein 3 (Rbm3)	32.23	66.77	1.05	2.39E−36
Zinc-alpha-2-glycoprotein (Azgp1)	19.98	65.02	1.70	3.70E−74
Lipoprotein lipase (Lpl)	18.8	60.49	1.68	1.51E−182
Extracellular calcium-sensing receptor (Casr)	11.78	24.42	1.05	3.92E−83
Solute carrier organic anion transporter family member 4C1 (Slco4c1)	7.23	21.74	1.58	2.14E−43
Bone morphogenetic protein receptor type-1B (Bmpr1b)	3.63	9.93	1.45	1.35E−21

### DAE increased the expression levels of positively regulated bone-related genes

Osteoporosis is mainly manifested as decreased bone mineral density. To study the protective mechanism of DAE on osteoporosis, we analyzed the differentially expressed genes. The results showed that DAE positively regulated bone development after treatment and significantly upregulated the expression levels of various genes that increase bone mineral density, including *S100a8, S100a9, Lcn2, Anxa1, Tf, Serpinb1a, Hp, Mpo, Pglyrp1,* and *Lcp1* (Table 4).

**Table 4:** The expression levels of genes related to bone (DAE vs. Blank)

Gene name	Blank (FPKM)	DAE (FPKM)	log2 fold change (DAE/Blank)	*p* value
Protein S100-A8 (S100a8)	33925.75	70037.03	1.04	0
Protein S100-A9 (S100a9)	19210.75	47459.88	1.30	0
Neutrophil gelatinase-associated lipocalin (Lcn2)	707.36	2701.88	1.93	0
Annexin A1 (Anxa1)	667.48	2700.59	2.01	0
Serotransferrin (Tf)	884.09	2481.53	1.49	0
Leukocyte elastase inhibitor A (Serpinb1a)	404.32	1621.5	2.00	0
Haptoglobin (Hp)	338.34	873.43	1.37	0
Myeloperoxidase (Mpo)	275.14	807.06	1.55	0
Peptidoglycan recognition protein 1 (Pglyrp1)	292.96	772.88	1.40	6.76E−234
Plastin-2 (Lcp1)	250.04	553.22	1.14	0

### DAE treats osteoporosis by regulating multiple genes based on the theory of “kidney governing bone”

According to the “kidney governing bone” theory of traditional Chinese medicine, we speculated that the effect of DAE on osteoporosis might be realized by regulating renal function. Our results showed that after DAE treatment, multiple genes were jointly upregulated or downregulated in kidney and bone, and that these genes play an important role in kidney and bone regulation, as shown in Tables 5 and 6.

**Table 5:** Significantly upregulated genes in both kidney and bone (DAE vs. Blank)

Gene name	Kidney				Bone			
	Blank (FPKM)	DAE (FPKM)	log2 fold change (DAE/Blank)	p value	Blank (FPKM)	DAE (FPKM)	log2 fold change (DAE/Blank)	*p* value
Heterogeneous nuclear ribonucleoproteins C1/C2 (Hnrnpc)	9.13	25.89	1.50	7.99E−38	11.12	39.01	1.81	4.55E−60
Fetuin-B (Fetub)	2.71	18.61	2.78	5.39E−54	1.57	12.47	2.99	8.71E−32
Type II inositol 1,4,5-trisphosphate 5-phosphatase (Inpp5b)	4.41	12.2	1.47	2.88E−25	8.49	18.34	1.11	5.18E−22
Probable peptide chain release factor C12orf65 homolog, mitochondrial (RGD1563482)	1.75	8.75	2.32	6.44E−16	4.43	10.65	1.26	4.86E−10
Leucine-rich repeat and coiled-coil domain-containing protein 1 (Lrrcc1)	1.36	3.68	1.44	2.73E−28	1.11	5.1	2.20	4.88E−22
Mitochondrial 2-oxodicarboxylate carrier (Slc25a21)	1.71	3.44	1.00	4.11E−05	1.99	4.76	1.26	2.15E−07
R-spondin-1 (Rspo1)	0.51	2.02	1.98	1.86E−06	8.67	19.01	1.13	2.11E−13
Transcription factor Sp4 (Sp4)	0.67	1.92	1.52	3.41E−07	2.09	4.87	1.22	4.36E−19
Muscleblind-like protein 3 (Mbnl3)	0.72	1.75	1.28	6.11E−09	2.63	10.19	1.95	1.57E−70
XK-related protein 8 (Xkr8)	0.01	1.53	7.25	6.33E−15	0.01	0.46	5.52	0.0003057
Protein piccolo (Pclo)	0.19	0.4	1.07	0.0001632	0.11	0.32	1.54	0.0001021

**Table 6:** Significantly downregulated genes in both kidney and bone (DAE vs. Blank)

Gene name	Kidney				Bone			
	Blank (FPKM)	DAE (FPKM)	log2 fold change (DAE/Blank)	p value	Blank (FPKM)	DAE (FPKM)	log2 fold change (DAE/Blank)	*p* value
H-2 class II histocompatibility antigen gamma chain (Cd74)	1028.42	469.35	− 1.13	0	471.62	222.24	− 1.08	8.15E−222
Hemoglobin subunit beta-2 (Hbb-b1)	315.47	150.66	− 1.07	3.10E−83	15850.76	6579.9	− 1.27	0
Creatine kinase B-type (Ckb)	281.88	108.66	− 1.38	1.35E−281	288.78	95.38	− 1.59	7.78E−297
Interferon alpha-inducible protein 27-like protein 2B (Ifi27)	216.8	97.44	− 1.15	2.51E−100	383.36	44.83	− 3.09616	0
Lymphocyte antigen 6E (Ly6e)	178.88	79.78	− 1.16	9.02E−120	128.48	27.52	− 2.22	1.08E−177
Bone marrow stromal antigen 2 (Bst2)	181.81	72.31	− 1.33	4.73E−88	253.63	62.72	− 2.02	3.31E−185
Rano class II histocompatibility antigen, B alpha chain (RT1-Ba)	149.69	69.71	− 1.10	1.90E−81	84.44	41.24	− 1.03	8.49E−36
Plasmalemma vesicle-associated protein (Plvap)	153.31	48.83	− 1.65	8.91E−278	29.52	8.59	− 1.78	4.84E−50
C-X-C motif chemokine 16 (Cxcl16)	60.51	28.52	− 1.08	1.30E−51	9.02	4.21	− 1.10	9.45E−08
Galectin-1 (Lgals1)	96.93	27.58	− 1.81	2.14E−42	1.13	0.12	− 3.24	1.24E−06
Interferon alpha-inducible protein 27-like protein 2B (Ifi27l2b)	63.39	27.41	− 1.20	1.62E−13	345.69	62.49	− 2.47	7.85E−157
Receptor activity-modifying protein 3 (Ramp3)	46.4	22.57	− 1.03	3.65E−26	32.75	12.51	− 1.39	2.30E−24
H-2 class I histocompatibility antigen, D-37 alpha chain (RT1-S3)	49.85	22.05	− 1.18	1.15E−76	87.38	41.29	− 1.08	2.78E−95
Neuronal regeneration-related protein (Nrep)	66.99	21.67	− 1.62	3.00E−77	21.61	10.29	− 1.07	3.07E−27
Endoglin (Eng)	43.95	21.03	− 1.06	4.10E−63	26.06	10.3	− 1.34	2.26E−44
Allograft inflammatory factor 1 (Aif1)	45.51	18.3	− 1.31	6.47E−19	2.38	0.75	− 1.67	4.69E−07
Complement C1q subcomponent subunit C (C1qc)	75.83	18.12	− 2.06	9.64E−95	207.25	82.69	− 1.32	6.67E−115
Complement C1q subcomponent subunit A (C1qa)	78.49	16.97	− 2.20	1.28E−102	315.83	104.33	− 1.60	4.19E−220
Transgelin (Tagln)	50.25	16.33	− 1.62	2.11E−49	24.15	11.6	− 1.06	1.51E−11
Fatty acid-binding protein, adipocyte (Fabp4)	82.44	15.78	− 2.38	1.02E−61	638.72	107.35	− 2.57	0
Interferon regulatory factor 7 (Irf7)	51.97	15.46	− 1.74	5.78E−108	333.62	22.01	− 3.92	0
Pleckstrin homology-like domain family A member 3 (Phlda3)	23.43	9.9	− 1.24	2.58E−22	23.62	4.58	− 2.37	23.62
SWI/SNF-related matrix-associated actin-dependent regulator of chromatin subfamily D member 3 (Smarcd3)	19.34	9.5	− 1.02	8.82E−17	3.31	1.15	− 1.52	8.31E−06
Complement C1q subcomponent subunit B (C1qb)	40.68	8.16	− 2.31	3.25E−64	120.82	49.9	− 1.28	5.64E−69
Ubiquitin-like protein ISG15 (Isg15)	24.08	7.8	− 1.62	1.37E−13	119.68	8.74	− 3.78	2.60E−133
Carbonic anhydrase 3 (Car3)		31.72	6.61	−2.26	1.65E-74	12.26	4.98	−1.30
MARCKS-related protein (Marcksl1)	15.45	6.51	− 1.24	1.29E−15	37.21	17.85	− 1.06	3.34E−23
Low affinity immunoglobulin gamma Fc region receptor IV (Fcgr3a)	12.21	5.67	− 1.10	2.84E−08	24.25	5.73	− 2.08	1.23E−29
T-cell surface glycoprotein CD4 (Cd4)	14.03	5.66	− 1.30	2.06E−17	37.75	14.18	− 1.41	8.04E−41
H-2 class I histocompatibility antigen, D-D alpha chain (RT1-N3)	17.65	5.24	− 1.75	6.31E−34	26.14	11.41	− 1.20	1.18E−23
Lysyl oxidase homolog 1 (Loxl1)	12.45	5.2	− 1.25	6.80E−21	16.47	7.04	− 1.23	1.29E−21
RT1 class I histocompatibility antigen, AA alpha chain (RT1-CE12)	10.25	5.03	− 1.02	3.98E−06	13.75	5.37	− 1.36	9.24E−10
Thyroid hormone-inducible hepatic protein (Thrsp)	26.19	4.86	− 2.42	2.38E−51	79.26	6.03	− 3.72	9.04E−189
Reticulocalbin-3 (Rcn3)	9.52	4.41	− 1.11	3.87E−08	55.94	19.44	− 1.52	3.49E−54
Guanine nucleotide-binding protein G(I)/G(S)/G(O) subunit gamma-T2 (Gngt2)	11.18	4.14	− 1.43	2.25E−05	61.97	19.47	− 1.67	8.62E−26
Complement factor D (Cfd)	21.54	4.12	− 2.38	7.85E−26	13.39	5.08	− 1.40	4.01E−05

### Validation of RNA-Seq data by qRT-PCR

**Fig. 3:**
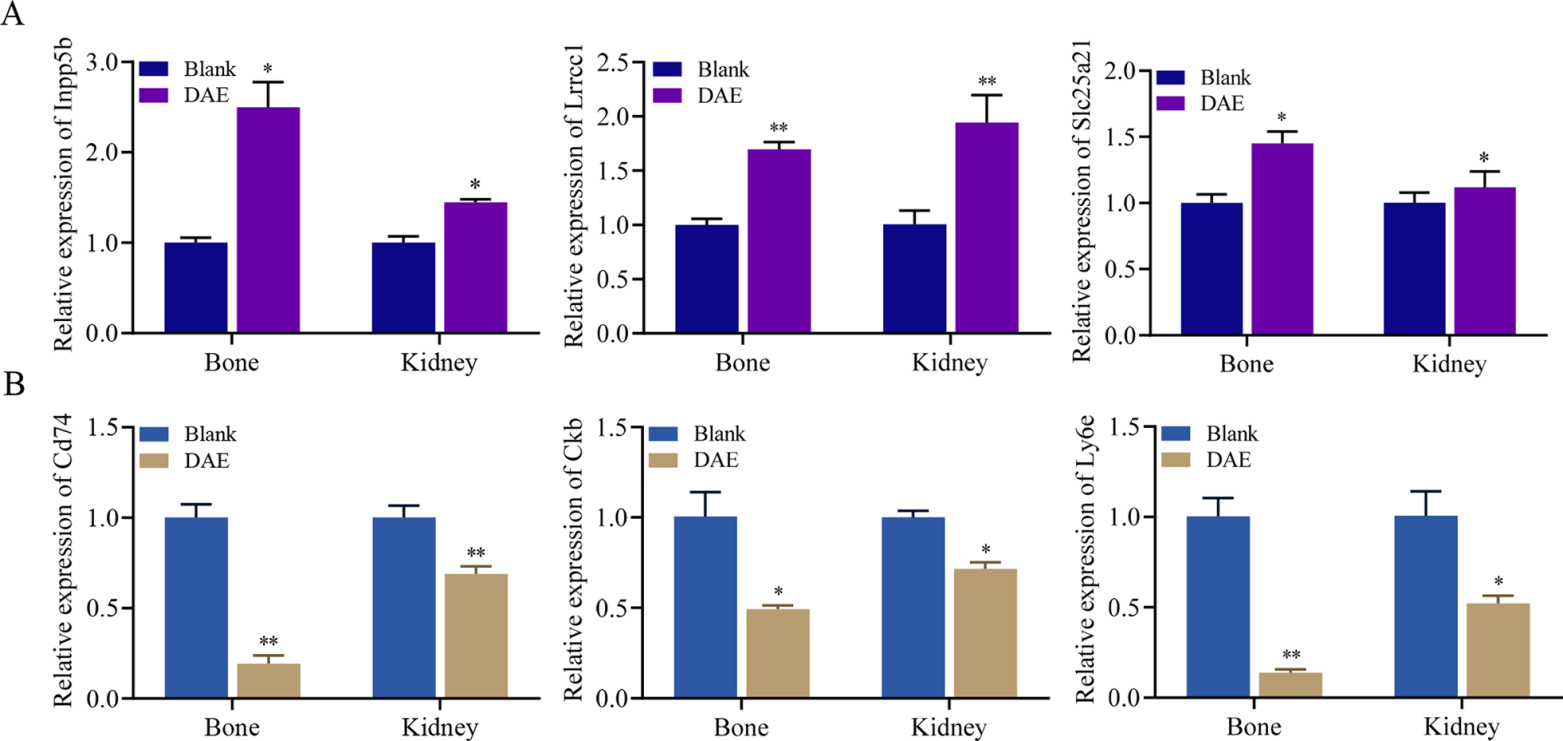
Verification of RNA-seq data by qRT-PCR assay. The relative mRNA levels of the DEGs were quantified by qRT-PCR. **A** Upregulated genes. **B** Downregulated genes. Data were presented as the mean with standard deviation for technical triplicate in an experiment representative of several independent ones. The asterisk *, **indicate significant differences using the Student’s *t*-test with *p* value < 0.05, < 0.01

We validated the expression levels of six randomly selected differentially expressed genes using the qRT-PCR method, including three significantly upregulated genes (*Inpp5b, Lrrcc1, Slc25a21*) and three significantly downregulated genes (*Cd74, Ckb, Ly6e*), which were significantly changed in both the bone and kidney. The specific primers used in this experiment are listed in Additional file1: Table S1. The relative fold change in each gene was normalized to the internal reference gene *RPL4*. The expression levels of the selected differentially expressed genes measured by qRT-PCR were consistent with the results of the RNA-Seq analysis, as shown in Fig. 3.

### DAE can promote bone trabecular formation and increase bone mineral density and bone volume fraction

**Fig. 4:**
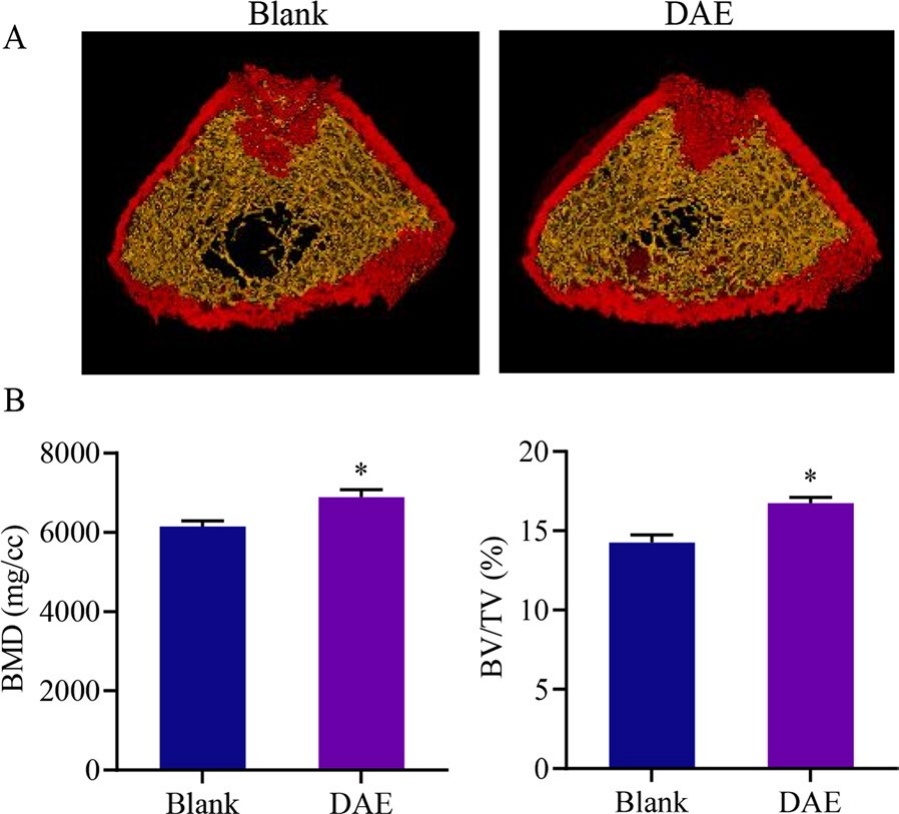
Micro-CT analysis of bone parameters. **A** Trabecular diagram of femur. **B** BMD and BV/TV analysis diagram. The asterisk *indicate significant differences using the Student’s *t*-test with *p* value < 0.05

To confirm the results of the RNA-seq analysis, after 21 days of intragastric administration of the DAE to SD rats, the skeletal effects of the DAE were analyzed using micro-CT. As shown in Fig. 4, compared with the negative control group (blank), DAE could increase the generation of trabecular meshwork in rats as well as the BMD and BV/TV in rats.

## Discussion

Deer antler has been considered a precious medicinal material for tonifying kidney and bone since ancient times. However, little is known about its role in treating kidney and skeletal system related diseases. In this study, we investigated the effects of DAE on kidney and bone using RNA-Seq technology combined with micro-CT.

In total, 1665 differentially expressed genes were identified in the kidney under DAE treatment, including 680 upregulated genes and 985 downregulated genes (DAE vs. blank). In the bone, following DAE treatment, 607 differentially expressed genes were identified, including 131 upregulated genes and 476 downregulated genes (DAE vs. blank). Based on GO enrichment analysis, the significantly enriched GO terms for the kidney transcriptome were mainly involved in development, structural molecular activity, and transportation, whereas the significantly enriched GO terms for the bone transcriptome were mainly involved in molecular regulation and structural molecule activity. According to KEGG enrichment analysis, the significant enriched pathways of the kidney transcriptome were mainly involved in the categories of cell adhesion molecules, hematopoietic cell lineage, the T cell receptor signaling pathway, osteoclast differentiation, and antigen processing and presentation. For bone, the significantly enriched pathways were mainly involved in the categories of cell adhesion molecules, the regulation of actin cytoskeletons, the Rap1 signaling pathway, the PI3K-Akt signaling pathway, osteoclast differentiation, hematopoietic cell lineage, ECM-receptor interaction, and the cell cycle. These results indicated that DAE regulates bone and kidney by regulating various functional genes and signaling pathways. These results suggest that DAE plays a role by regulating renal homeostasis and improving bone mineral density, which also verifies the “kidney governing bone” theory at the molecular level.

The results show that genes involved in regulating kidney function and homeostasis were significantly upregulated, including *Slco1a6, Resp18, Tmigd1, Rbm3, Azgp1, Lpl, Casr,* and *Slco4c1*. Targeted disruption of the Resp18 locus in the SD rat increases salt-induced hypertension and associated renal damage [18]. TMIGD1 is a cell adhesion molecule that is expressed primarily by intestinal and renal epithelial cells and acts as an antioxidant stress protective agent [19]. RBM3 is closely related to the proliferation rate of human embryonic kidney (HEK293) cells, Downregulation of RBM3 inhibits cell proliferation and finally leads to cell death [20]. AZGP1 is a secreted protein synthesized by epithelial cells, which exerts antifibrotic effects in the kidney [21]. LPL is a lipoprotein lipase that is protective in patients with chronic kidney disease with impaired plasma VLDL lipolysis [22]. CaSR is a G protein-coupled receptor that plays a key role in renal calcium homeostasis [23]. SLCO4C1 is an organic anion that transports polypeptides whose transporter eliminates uremic toxins and attenuates hypertension and renal inflammation [24, 25]. BMPR1B plays a key role in renal signal transduction and protection against renal fibrosis [26].

The results showed that genes involved in the positive regulation of the skeletal system, including *S100a8, S100a9, Lcn2, Anxa1, Tf, Serpinb1a, Hp, Mpo, Pglyrp1,* and *Lcp1*, were significantly upregulated. S100A8 has been shown to be associated with osteoblast differentiation, and both S100A8 and S100A9 may contribute to calcification of the cartilage matrix, its replacement with trabecular bone, and the redox regulation in bone resorption [27]. LCN2 in bone can regulate key secretory factors and cytokines to alter the BM microenvironment, thus regulating the bone marrow microenvironment and promoting bone regeneration [28]. Annexin 1 is a 37 kDa protein, the loss of which causes abnormal skull development in mice [29]. Tf assists in transporting iron to the bone marrow for hemoglobin synthesis [30]. Serpinb1a regulates bone homeostasis by inhibiting osteoclast formation and osteoblast differentiation [31]. Hp is a member of the acute phase proteins, and deletion of the Hp gene results in significant bone loss with increasing osteoclast formation [32]. MPO is a heme peroxidase, which plays a protective role in bone turnover by limiting osteoclastogenesis and bone resorption physiologically by modulating the intracellular H_2_O_2_ concentration [33]. Peptide 17.1A is capable of reducing periarticular inflammation, inhibiting the development of synovitis, and exhibiting a protective effect on cartilage and bone tissues [34]. Lcp1 is a calcium-binding protein that regulates intracellular calcium by storing and releasing Ca^2+^, it coordinate the regulation of intracellular Ca^2+^ during osteoblast differentiation [35].

We analyzed the differentially expressed genes with consistent patterns in both the kidney and bone, of which 11 genes were significantly upregulated and 19 genes were significantly downregulated following treatment with DAE. We further demonstrated that DAE may also play roles in regulating kidney and bone functions by modulating functional gene expression patterns that might be related to the “kidney governing bone” theory. Finally, micro-CT verifies that DAE could increase the generation of trabecular meshwork in rats as well as the BMD and BV/TV in rats. Taken together, we have currently discovered some clues regarding the molecular mechanisms of DAE in regulating bone development, which might be governed by kidney function. These results deepen the current knowledge about the molecular effects of DAE on bone and kidney regulation. Furthermore, this study may provide possible strategies to further prevent and treat diseases using Traditional Chinese Medicine following the “kidney governing bone” theory. However, other in vitro and in vivo approaches still need to be well designed and performed, such as proteomic and histological analyses, as well as gain- and loss-of function analyses, to fully dissect the underlying mechanisms of DAE on bone and kidney regulation based on the “kidney governing bone” theory.

## Conclusion

This study has deepened the current knowledge about the molecular effects of DAE on bone and kidney positive regulation. Our findings are consistent with the “kidney governing bone” theory, which has been widely used in clinical practice for thousands of years. Additionally, this study may provide a possible strategy for the further application of the kidney-bone theory in the prevention and treatment of joint diseases with Traditional Chinese Medicine formulations.

## Supplementary Information

**Additional file1**. **Table S1**: List of primers used for qRT-PCR validation.

## Data Availability

The datasets used and/or analyzed during the current study are available from the corresponding author on reasonable request.
