# Supplementary material for: Transcriptional sequencing analysis reveals the potential use of deer antler for “tonifying the kidney and strengthening bone”
Source: J Orthop Surg Res. 2022 Sep 14;17:419. doi: 10.1186/s13018-022-03308-w (PMC9476563; doi:10.1186/s13018-022-03308-w)
Supplement: Supplementary file 1 — Additional file1. Table S1: List of primers used for qRT-PCR validation. [file 13018_2022_3308_MOESM1_ESM.docx]

Table S1 List of primers used for qRT-PCR validation

| Gene name | Primer | Sequence |
| --- | --- | --- |
| Inpp5b | Forward primer  Reverse primer | TATACACACCGGAGGATGGC CTGGTGTGTGAACCAAAGGG |
| Lrrcc1 | Forward primer  Reverse primer | ACAACACTTACCGGTCCCTT  GATTGCCTGGCTGTGAACAT |
| Slc25a21 | Forward primer  Reverse primer | GGACAGTGGGCTCAGTCTTT  AAGCAGCCTCGGTACTTGAT |
| Cd74 | Forward primer  Reverse primer | AGTCTGGACCCGTGAACTAC  CCAGTGGCTCTTTAGGTGGA |
| Ckb | Forward primer  Reverse primer | CATCATGACAGTGGGTGCAG  TGTCTGGGTTGAGGTCAGTC |
| Ly6e | Forward primer  Reverse primer | CTGATGTGCTTCTCCTGCAC  TTCAGGGTGTAGCCAAGGTT |
